# Paternal Smoking and Risk of Attention Deficit Hyperactivity Disorder in Children: A Systematic Review and Meta-Analysis

**DOI:** 10.1192/j.eurpsy.2025.372

**Published:** 2025-08-26

**Authors:** B. Dachew, Y. Damtie, G. Ayano, R. Alati

**Affiliations:** 1 School of Population Health, Curtin University, Perth; 2 Institute for Social Sciences Research, The University of Queensland, Brisbane, Australia

## Abstract

**Introduction:**

Attention Deficit Hyperactivity Disorder (ADHD) is a prevalent neurodevelopmental disorder that significantly affects children’s behaviour, attention, and academic performance. While the impact of maternal smoking during pregnancy on ADHD risk is well-established, emerging research suggests that paternal smoking may also contribute to this risk. However, the relationship between paternal tobacco use and ADHD remains underexplored, with existing studies presenting mixed results.

**Objectives:**

This systematic review and meta-analysis aim to clarify the extent of this association and provide a comprehensive assessment of the evidence available.

**Methods:**

All relevant studies in CINAHL, Embase, PsycINFO, PubMed, Scopus, and Web of Science databases were searched from inception until 15 March 2024. Both conventional and cumulative meta-analyses were conducted. Pooled odds ratios with 95% confidence intervals (CIs) were calculated using a random-effects model. The heterogeneity among studies was assessed using the I^2^ test, and the presence of small study effects was evaluated using funnel plots and Egger’s test. Sensitivity and subgroup analyses were also performed.

**Results:**

Twenty observational studies involving over 294, 236 study participants from 16 different countries were included. We found that paternal smoking was associated with a 22% increased risk of ADHD in children (RR=1.22, 95% CI: 1.12, 1.33). The observed association has remained stable since 2014, with minimal fluctuations in effect sizes and their corresponding 95% CIs. Our subgroup analysis revealed that this association is only evident among studies that did not account for maternal smoking (OR=1.23, 95% CI: 1.10, 1.38, *n*=8), while no increased risk of ADHD was found in studies that adjusted for maternal smoking (OR=1.14, 95% CI: 0.98, 1.33), suggesting that maternal smoking may confound the observed association.

**Image 1:**

**Figure:**
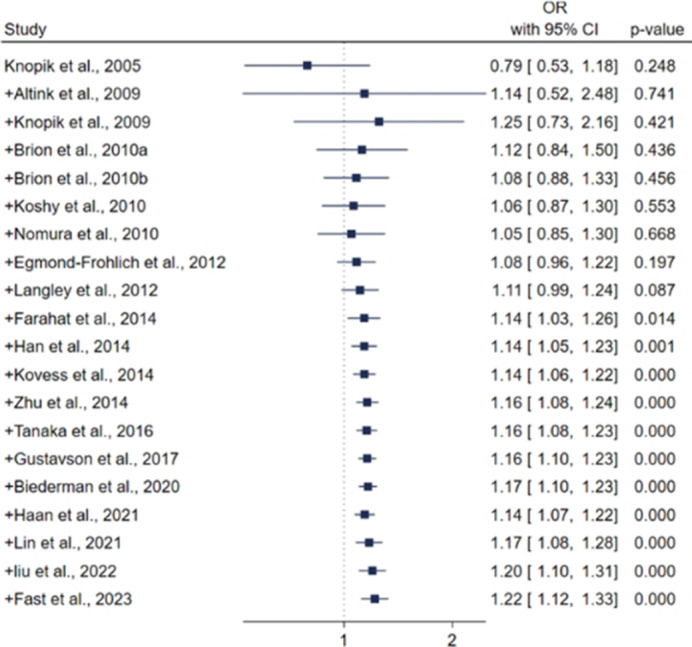

**Conclusions:**

Paternal smoking may increase the risk of ADHD in children. Future studies should focus on maternal and paternal comparisons to disentangle the independent and combined effects of parental smoking on ADHD risk in children.

**Disclosure of Interest:**

None Declared

